# Everyday discrimination and satisfaction with nature experiences

**DOI:** 10.3389/fepid.2024.1212114

**Published:** 2024-05-30

**Authors:** Leah H. Schinasi, Jourdyn A. Lawrence

**Affiliations:** ^1^ Department of Environmental and Occupational Health, Drexel University, Philadelphia, PA, United States; ^2^ Urban Health Collaborative, Drexel University, Philadelphia, PA, United States; ^3^ Department of Epidemiology and Biostatistics, Drexel University, Philadelphia, PA, United States

**Keywords:** green space, nature-based solutions, everyday discrimination, discrimination, parks

## Abstract

**Introduction:**

There is growing interest in creating public green spaces to promote health. Yet, discussions about these efforts often overlook how experiences of chronic discrimination—which may manifest as racism, sexism, or homophobia, and more—could undermine satisfaction with nature experiences.

**Methods:**

Using data from the 2018 wave of the National Opinion Research Center (NORC) General Social Survey (GSS), we quantified associations of frequency of everyday discrimination, operationalized using the Everyday Discrimination Scale (EDS, the primary independent variable), with respondents' perceptions of nature experiences and with their reported time spent in nature. Specifically, we quantified associations with the following three variables: (1) dissatisfaction with day-to-day experiences of nature, (2) not spending as much time as they would like in natural environments, and (3) usually spending at least one day per week in nature. We used survey-weighted robust Poisson models to estimate overall associations, and also stratified analyses by racial/ethnic and gender identity categories.

**Results:**

Of 768 GSS respondents, 14% reported dissatisfaction with nature experiences, 36% reported not spending as much time as they would like in nature, and 33% reported that they did not spend at least one day per week in nature. The median non-standardized EDS, coded such that a higher value indicates greater frequency of discrimination, was 11 (interquartile range: 8, 15). Prevalence of reporting dissatisfaction with day-to-day experiences in nature was 7% higher in association with every one unit increase in EDS score above the median (PR: 1.07, 95% CI: 1.02–1.11). The prevalence of reporting not spending as much time as one would like in nature was 2% higher for every unit increase in higher than median everyday discrimination frequency (PR: 1.02, 95% CI: 1.00–1.05). Higher than median frequency in everyday discrimination was not associated with spending less than one day per week in nature. Race/ethnicity and gender identity did not modify associations.

**Conclusion:**

Greater frequency of everyday discrimination is associated with less satisfaction with experiences in nature. This relationship could undermine efforts to promote health equity through green interventions.

## Introduction

Across the world, cities are investing in green infrastructure—planting trees, creating parks, and more—to promote population and planetary health (1, 2). These initiatives are supported by mounting evidence that green space availability supports well-being and health equity (3, 4). Scholars posit that green spaces can promote health through a myriad of pathways, such as by enhancing physical activity (5), social cohesion (6), and immune function (7). Other pathways include mitigating environmental hazards [e.g., storm water (8), heat (9, 10), noise (11)] and reducing stress (12, 13). Yet, to date, most environmental health research has overlooked the impacts of sociocultural contexts and personal identities (e.g., race and gender) on green space use and satisfaction. Without consideration of these contexts, green space creation may fail in its goals to support health and justice.

Systems and structures of inequities and discrimination have impacted the spatial distribution of green spaces, as well as perceptions and patterns of their use. For example, more vegetation is often found in wealthier and Whiter neighborhoods than in poorer, minoritized communities (14–17). This is a situation of distributive injustice (18), which has been linked to historical systems of racism and marginalization, including mortgage discrimination (19, 20) and Jim Crow era separate-but-equal policies (21). Early efforts to create, manage, and conserve parks were instilled with eugenic and racist ideologies; the “great outdoors” was conceptualized by and for cisgender White American men (22). Indigenous knowledge was ignored when decisions about natural resource management and regulation were made (23), and park creation efforts perpetuated the exclusion of minoritized groups (22). Leadership, practices, and policies concerning park creation and management resulted in minimal park and outdoor space for marginalized people to enjoy safely. Despite the passage of the Civil Rights Act in 1964, exclusionary and discriminatory practices, including policing and enforcement of racial boundaries and racially restrictive covenants persist (24).

The intermingling of these policies with historical and contemporary violence may have important implications for green space quality, availability, and accessibility (16, 24–26). These practices may also overlap with experiences of everyday discrimination, defined as subtle and chronic forms of discrimination that are directed against those holding marginalized identities (27), including ones defined by race, ethnicity, gender, sexual orientation, age, socioeconomic position, and disability status. Everyday discrimination manifests in different ways. For example, in the case of gender identity, everyday discrimination may manifest as naive assumptions about a woman's preferences or abilities; their work or caretaking roles, or by offering different gender-stratified opportunities (27). Racialized everyday discrimination may be subtle and indirect and manifest in ways such as increased surveillance or expectations about intelligence or preferences (27).

Everyday discrimination may lead to feelings of exclusion or dissatisfaction in public natural spaces (27, 28). Prior experiences of everyday discrimination may cause individuals to limit social interactions (29); this may include limiting time in public parks or natural spaces (30). Women have been found to be less represented in public parks as compared to men (28, 31, 32), potentially explained by concerns over safety or harassment in public green spaces (33). Social and cultural norms and expectations around gender roles, such as family caregiving and prioritizing the needs of others, may also explain more frequent park use among men than women (34). For example, in one study, women noted that, beyond work and family care responsibilities, they had little time left for personal activities such as park visitation (34). Transgender or non-binary individuals may avoid visiting public green spaces because of concerns over safety. They may also change their behaviors in anticipation of harassment and discrimination (e.g., preparing an exit strategy, altering their usual clothing), though in some cases, they may actively resist and exist despite these concerns (35, 36). Perceptions of discrimination and exclusion, fear of crime, or policing (33) could also explain under-representation of racially or ethnically minoritized groups (22, 24, 37). The intersection of multiple marginalized identities may intensify these strategies (27, 32, 36, 38). Yet, there has been little consideration of how sociocultural contexts and chronic experiences of oppression give rise to inequities in green exposures, experiences, and perceptions. Failure to address and acknowledge these critical questions may unintentionally perpetuate and reinforce inequities and cause public health and environmental health scientists to fall short in their antiracist and social justice commitments (29, 39–43).

We leveraged data from the National Opinion Research Center (NORC) General Social Survey (GSS) of American households to quantify associations between everyday discrimination and satisfaction with experiences in nature. In 2018, a subset of GSS participants provided data on perceptions of nature experiences and chronic discrimination. We hypothesized that respondents who reported high levels of chronic discrimination in everyday life would also be dissatisfied with their experiences and time spent in nature. This investigation responds to recent calls for environmental health researchers to explicitly consider the role of social conditions and systems of marginalization in creating and perpetuating environmental exposures and health inequities (40).

## Methods

### Study design and data

We conducted a cross-sectional study using data from the 2018 wave of the National Opinion Research Center (NORC) General Social Survey (GSS) administered at the University of Chicago. The GSS is a biannual, household-based, multistage cluster-sampled survey of a representative sample of non-institutionalized adults ages 18 and older in the continental United States. Computer assistant personal interviews were conducted with the 2018 survey participants (*N* = 1,173, response rate = 59.5%) (39, 44). After the computer-assisted personal interviews were conducted, GSS participants responded to additional questions in a self-administered questionnaire (45). The GSS administers questions in three different “ballot” forms; some questions are unique to a single ballot. Because respondents do not have to respond to the complete survey, the three-tiered ballot design reduces respondent burden. We limited our analysis to Ballot 3 respondents (46), which contains the subsample that responded to questions about experiences in nature (i.e., public parks, gardens, or trails). The original GSS was approved by the IRB of the University of Chicago. Given the use of publicly available and deidentified data from the GSS in the present analysis, it is considered non-human subjects research and did not require additional IRB approval.

### Everyday discrimination

We operationalized everyday discrimination using the abbreviated version of the Everyday Discrimination Scale (EDS), administered by GSS investigators (42). The abbreviated EDS includes five items that assess frequency of day-to-day experiences of interpersonal discrimination. GSS participants responded to the following: In your day-to-day life, how often have any of the following happened to you: (1) You are treated with less courtesy or respect than other people; (2) You receive poorer service than other people at restaurants or stores; (3) People act as if they think you are not smart; (4) People act as if they are afraid of you; (5) You are threatened or harassed. For each item, participants selected one of the following options: (1) Almost every day, (2) once/week, (3) a few times per week, (4) a few times per year, (5) less than once a year, or (6) never. We calculated a single EDS by reverse coding the scores and summing them, such that a higher value represents greater frequency of discrimination. After this coding, the maximum possible unstandardized EDS value is 30. This score would represent the highest possible frequency of discrimination. We also calculated a standardized EDS by *z*-score standardizing the reverse-coded responses to each question and summing the *z*-scores together.

### Nature experiences

Three variables representing perceived experiences and time spent in nature served as the primary dependent variables. These variables were ascertained from the following responses to questions in the 2018 wave of the NORC GSS: (1) I am satisfied with my day-to-day experience of nature; (2) I spend as much time as I would like in natural environments; and (3) Usually, I spend time in natural environments, such as public parks, gardens or trails, at least once a week. Response options were the following: (1) strongly agree, (2) somewhat agree, (3) somewhat disagree, and (4) strongly disagree. We created binary variables, coded 1 = somewhat disagree/strongly disagree, 0 = strongly agree/somewhat degree.

### Additional variables

We created a three-level marginalized gender identity variable using responses to the following question asked of participants in the self-administered questionnaire version of the 2018 GSS: “What is your current gender?” Response options (using GSS terminology) were the following: (1) “woman”, (2) “man”, (3) “transgender”, and (4) “a gender not listed here”. We combined the woman (*n* = 375), transgender (*n* = 1), and other/not listed (*n* = 1) categories to create a variable capturing marginalized gender identities (compared to those who identified as a man). Of the 789 respondents of the 2018 GSS Ballot 3, 83 were missing responses to this question. We created a third, missing gender category for these 83 Ballot 3 respondents. We coded race/ethnicity using self-reported race from the 2018 GSS. To account for small cell counts within individual categories, we created a binary variable, coded 1 to indicate having a marginalized racial or ethnic identity (Black/African American, American Indian/Alaska Native, Asian Indian, Chinese, Filipino, Japanese, Korean, Vietnamese, Other Asian, Native Hawaiian, Guamanian or Chamorro, Samoan, Other Pacific Islander, other race, Hispanic) and 0 to represent having a privileged identity (i.e., non-Hispanic White identifying individuals). Five of the Ballot 3 participants were missing data on self-reported racial/ethnic identity; we excluded these five people from the analysis.

To capture occupational status of the GSS sample, we created a categorical variable from the following question: “Last week, were you working full-time, part-time, going to school, keeping house, or what?” We coded the categorical variable as follows: (1) Working for pay, part or full time; (2) Unemployed, retired, not working due to illness/vacation/strike; (3) In school; (4) Keeping house; and (5) Other.

### Analysis

We used survey weights provided by GSS investigators to account for the complex survey design. In analyses, we ran separate models for each of the primary dependent variables, each coded as a binary term; 1 indicated dissatisfaction with nature experiences, inability to spend as much time as they would like in nature, or spending <1 day per week in nature, and 0 indicated satisfaction with nature experiences, ability to spend as much time as they would like in nature, and usually spending at least once per week in nature. The independent variable in all analyses was the standardized EDS score. Because nearly every member of a marginalized population likely experiences some form of discrimination, a reference value of zero may not reflect an absence of experiences of discrimination. Indeed, studies have found that, compared to those who report experiences of discrimination, those who report no discrimination experience worse or similar levels of adverse health outcomes (47–50). For example, in one study, Black women who reported that they usually tolerated unjust treatment had over four-fold higher prevalence of hypertension as compared to Black women who respondents who responded (47). Others have found that marginalized identities deny having experienced discrimination, even when it has occurred (51). These studies have posited other processes affecting the report of discrimination, like inability to perceive discrimination at the personal level, suppression of reactions to discrimination due to societal expectations and restrictions, avoidance of discomfort owing to recognition of having been the target of discrimination, or internalization of unfair treatment (47–50). Thus, we operationalized the EDS by centering the standardized score at the median of the distribution in the Ballot 3 sample. We modeled the median-centered EDS score as a linear term. We identified linear coding as optimal by running and comparing sets of models with the variable coded as a natural cubic spline term with 3 and 2 degrees of freedom, and then again coded as linear. We identified the parameterization that was associated with the smallest AIC statistic, taking into account all three dependent variables (Supplementary Materials S1). We also preferred a linear parameterization because it simplifies translation of results.

Because the three dependent variables in this analysis were binary and prevalent, we attempted to estimate associations between the EDS and the three nature experience variables using log-binomial models. Log binomial, rather than logistic regression models, are appropriate in contexts such as this with a common outcome. By contrast, logistic models overestimate prevalence or risk ratios (52). However, the log-binomial models failed to converge. Thus, we used survey-weighted Poisson regressions with robust variance to estimate associations, which are an appropriate alternative to log-binomial approaches (53–55).

We developed a directed acyclic graph (DAG) to conceptualize relationships among measured and unmeasured confounders and identify a minimally sufficient adjustment set for the analyses. In addition, the DAG illustrates hypotheses about the ways by which marginalized identities might be related to green space availability, nature experiences, and everyday discrimination (56) (Supplementary Materials S2). As depicted in the DAG, we conceptualize race as a social construct that is the product of historical and present-day processes, policies, and practices. We consider individual-level race/ethnic identity as a variable that captures people who, because of social, cultural, economic, and/or historical processes and systems (56, 57), may experience high levels of everyday discrimination. We conceptualize gender identity as a social construct that has implications for experiences of discrimination, and for gendered norms and cultural expectations (58). Based on the DAG and *a priori* hypotheses, we adjusted all models for working status (coded working part time/full time for pay, unemployed/retired, keeping house, in school, other), US Census region (categories presented in Table 1), and age.

**Table 1:** Survey weighted descriptive statistics for ballot 3 respondents included in the analysis.

Variable	Median and interquartile range or proportion (*N* = 768)	SE
Age (years)	40 (28, 58)	1.26^a^
Discrimination scale, standardized	−0.47 (−2.6, 2.16)	0.13^a^
Discrimination scale, unstandardized	11 (8,15)	0.25^a^
Gender identity^b^		
Man	0.42	0.02
Marginalized gender identity	0.50	0.02
Not reported	0.08	0.01
Racial/ethnic identity		
Non-Hispanic White	0.73	0.02
Non-Hispanic Black	0.16	0.01
Other	0.06	0.01
Hispanic/Latinx	0.05	0.01
Employment status		
Working for pay	0.61	0.02
In school	0.06	0.01
Keeping house	0.10	0.01
Other	0.02	0.01
Unemployed, not working due to illness/vacation/strike, or retired	0.22	0.02
Region		
Eastern North Central	0.16	0.02
Eastern South Central	0.07	0.02
Middle Atlantic	0.09	0.01
Mountain	0.06	0.02
New England	0.06	0.02
Pacific	0.17	0.02
South Atlantic	0.22	0.03
Western North Central	0.04	0.01
Western South Central	0.13	0.02
Satisfied with experience in nature		
Yes	0.86	0.02
No	0.14	0.02
Spend as much as time would like in nature		
Yes	0.64	0.02
No	0.36	0.02
Spend at least one day per week in nature		
Yes	0.67	0.02
No	0.33	0.02

SE, standard error.

^a^ Standard error is associated with the median of the distribution.

^b^ Respondents were asked to report their current gender identity and given the options of man, woman, transgender, or other gender not listed. Here, the marginalized gender identity category includes respondents who reported their gender identity as either woman, transgender, or “other/not listed”.

We explored effect measure modification of associations by categories of racial/ethnic and gender identity. To do so, we reran models with additional inclusion of interaction terms between the EDS score term and either gender or racial/ethnic identity (as well as single covariates gender or racial/identity). While we recognize that individuals hold multiple identities, we did not have sufficient statistical power to explore modification by intersectional identities. We quantified statistical evidence of heterogeneity across strata of the modifiers by conducting regression-based likelihood test of interactions, comparing nested models with and without interaction terms (59).

From all model output, we interpret the exponentiated Beta coefficients as the change in the prevalence of the nature experience variable associated with every one-unit increase in higher than median everyday discrimination frequency. All analyses were conducted using the survey package in R (Version 4.2.2) (60, 61).

## Results

Table 1 presents descriptive statistics on the study population. GSS Ballot 3 participants were located across the United States, with the largest proportions living in the South Atlantic, Pacific, and Eastern North Central Census regions. The median age of participants was 40 years [Interquartile Range (IQR): 28, 58]. A slightly higher proportion of participants identified as a marginalized gender identity than as a man (Proportions: 0.50 and 0.42, respectively). Most participants identified as White (Proportion: 0.73) and were working for pay (either part or full time, Proportion: 0.61). Many participants said that they were satisfied with their time in nature, that they experience as much time as they would like in nature, and that they spend at least one day per week in nature (Proportions: 0.86, 0.64, 0.67, respectively**)**. The median of the unstandardized EDS score, coded such that higher values indicate greater frequency of discrimination was 11 [Interquartile Range (IQR): 8–15, Table 2]. The unstandardized mean of frequency of everyday discrimination was modestly higher among people who reported being a member of a marginalized racial group as compared to non-Hispanic White (Mean: 11.9, SE: 0.4 for individuals with marginalized racial/ethnic identities, Mean: 11.6, SE: 0.2 for non-Hispanic White individuals) and the median was the same (11). Everyday discrimination was reported to occur more frequently by respondents who identified as men (Mean of the unstandardized score: 11.9, SE: 0.3) as compared to participants who identified as members of marginalized gender groups (Mean of unstandardized score: 11.6, SE: 0.3).

**Table 2:** Distribution of the standardized and unstandardized everyday discrimination score, reverse coded such that higher values indicate more frequent every discrimination.

	Unstandardized		Standardized	
	Median (IQR)	Mean (SE)	Median (IQR)	Mean (SE)
Overall	11 (8, 15)	11.6 (0.2)	−0.47 (−2.57, 2.16)	0.16 (0.13)
Race/ethnicity				
Non-Hispanic White race/ethnicity	11 (8,14)	11.6 (0.2)	−0.47 (−2.44, 1.99)	0.11 (0.15)
Hispanic, Black, and other race/ethnicities	11 (8, 15)	11.9 (0.4)	−0.42 (−2.68, 2.31)	0.26 (0.30)
Gender identity				
Identified as a man	11 (9, 15)	11.9 (0.3)	−0.21 (−1.88, 2.44)	0.31 (0.19)
Identified as a woman, trans, or other gender identity	11 (8, 14)	11.6 (0.3)	−0.51 (−2.62, 2.00)	0.11 (0.21)
Did not report a gender identity	9 (7, 14)	11.0 (0.5)	−1.52 (−3.09, 1.97)	−0.36 (0.40)

IQR, interquartile range; SE, standard error.

### Association between everyday discrimination and nature experiences

Prevalence of dissatisfaction with nature experiences was 7% higher in association with every one unit increase in frequency of everyday discrimination above the median (PR: 1.07, 95% CI: 1.02–1.11, Table 3). The prevalence of reporting not spending as much time as one would like in nature was 2% higher in association with every one-unit increase in the frequency of everyday discrimination above the median (PR: 1.02, 95% CI: 1.00–1.05). Frequent everyday discrimination beyond the median was not associated with spending less than one day per week in nature (PR: 1.00, 95% CI: 0.97–1.04). Neither race nor gender identity modified associations.

**Table 3:** Prevalence ratio (PR) estimates of associations between everyday discrimination and poor nature experiences, overall and stratified by marginalized gender and racial/ethnic identities^a^.

	PR	LL	UL	*p* for LRT
Not satisfied with experience in nature				
Overall	1.07	1.02	1.11	
*Racial/ethnic identity*				
Non-Hispanic White	1.08	1.03	1.14	0.4
Marginalized racial/ethnic group	1.03	0.93	1.13	
*Gender identity*				
Men	1.02	0.95	1.10	0.4
Marginalized gender identity	1.09	1.03	1.15	
Gender identity not given	1.18	0.90	1.56	
Do not spend as much time as you want in nature				
Overall	1.02	1.00	1.05	
*Racial/ethnic identity*				
Non-Hispanic White	1.03	1.00	1.06	0.6
Marginalized racial/ethnic group	1.02	0.96	1.07	
*Gender identity*				
Men	1.01	0.97	1.05	0.7
Marginalized gender identity	1.04	1.00	1.07	
Gender identity not given	0.97	0.88	1.07	
Do not spend at least one day per week in nature				
Overall	1.00	0.97	1.04	
*Racial/ethnic identity*				
Non-Hispanic White	0.99	0.94	1.04	0.2
Marginalized racial/ethnic group	1.04	0.99	1.09	
*Gender identity*^b^				
Men	1.01	0.96	1.06	0.3
Marginalized gender identity	1.01	0.97	1.07	
Gender identity not given	0.91	0.80	1.04	

PR, prevalence ratio; LL, lower limit; UL, upper limit, LRT, likelihood ratio test.

^a^ PR estimates are derived from survey-weighted Poisson regression models with robust standard errors. Models are adjusted for occupational status, age, and US Census region. The primary independent variable was the everyday discrimination score, standardized and centered at the median of the distribution. PR estimates can be interpreted as the incremental increase in prevalence associated with every one-unit increase in the everyday discrimination score above the median of the distribution of the score within the study population.

^b^ Respondents were asked to report their current gender identity and given the options of man, woman, transgender, or other gender not listed. Here, the marginalized gender identity category includes respondents who reported their gender identity as either woman, transgender, or “other/not listed”.

## Discussion

In this analysis of a nationally representative sample of U.S. adults, a higher proportion of respondents reporting higher frequency of everyday discrimination also reported low levels of satisfaction with their day-to-day experiences in nature, relative to respondents who reported lower frequency of discrimination. Higher frequency of discrimination was also modestly associated with respondents' reporting that they did not spend as much time as they would like in nature. However, more frequent discrimination was not associated with absolute amount of time spent in nature, assessed using respondents’ self-reports of spending time in nature at least once per week. These results suggest that everyday discrimination has greater implications for *subjective* satisfaction with nature experiences, rather than with ability to spend at least one time per week in nature.

From this study, we cannot ascertain if respondents' reports of experiences of discrimination occurred in natural settings, nor can we establish a causal link between having experienced discrimination in a natural setting and subjective satisfaction with nature experiences. In fact, some may seek out nature experiences as a refuge (62). It is possible, however, that at least a few of the reported experiences of discrimination occurred when respondents were visiting nature. There have been reports of such incidents in the media. For example, in 2020, Amy Cooper, a White woman walking her dog without a leash in Central Park, made false claims to the police of violence from Christian Cooper, a Black birdwatcher, after he asked that she leash her pet per the park rules (37). Experiences of everyday discrimination have been documented in other public spaces, including metro platform benches in Milan, Italy (63).

Racism manifests at institutional, personally mediated, and internalized levels (64). These levels are interrelated (65) and may reinforce one another to produce adverse outcomes (66), including dissatisfaction with nature experiences. For example, qualitative interviews with Black community members living near Cedar Hill State Park, Texas revealed that prior experiences of racialized discrimination, perceptions of the public park as a “White space”, and a long history of institutional racism that resulted in segregation from public spaces contributed to negative perceptions of the park (67). The lower levels of satisfaction with nature experiences, expressed by respondents who reported greater everyday discrimination, may be explained, at least in part, by a lack of amenities [e.g., picnic tables, shade trees, drinking fountains (68)] or a preponderance of “disamenities” (e.g., litter, crime) (69, 70) in green spaces most accessible to them. Indeed, racialized, ethnic, or income based spatial disparities in the prevalence of positive (or negative) park characteristics have been observed in some, though not all, geographic areas (71–73). The lack of amenities, or the preponderance of “disamenities”, could undermine satisfaction with nature experiences. For example, in a study based in the Netherlands, higher availability of green spaces was associated with greater satisfaction with one's neighborhood. However, the association was mediated by self-reported green space quality (74).

Race/ethnicity is only one identity that may contribute to experiences of discrimination. There is a small body of evidence suggesting that fear makes women modify their behaviors in urban settings (75, 76) and contributed to reduced park use among women and marginalized racial/ethnic groups (77). Research has also shown that green interventions contribute to feelings of fear in women (32) but less so among men (78). Also, those who identify as transgender or non-binary have been shown to avoid public green spaces because of concerns over safety or harassment (35, 36).

Creation of green spaces has been posited to be a promising intervention to support health and well-being (79). However, previous research has suggested that satisfaction with local green spaces is a more important predictor of well-being than its availability or use (80). However, in our analysis, we found that a higher proportion of individuals reporting more frequent everyday discrimination also reported modestly lower levels of satisfaction with their experiences in nature. Thus, everyday discrimination and its association with satisfaction with nature experiences may limit green spaces in their full potential for health promotion.

We acknowledge several limitations in this analysis. First, because of a relatively small sample size, we were underpowered to explore effect measure modification of associations by intersectional marginalized identities. Also, we acknowledge two limitations related to the coding of gender identity. First, the GSS included transgender as a separate category from man and woman, rather than including this with the self-identified gender category and adding a non-binary option (i.e., men, women, or non-binary) (45). Second, based upon the methods used to collect gender, we grouped together what we assume are cis-gendered women and transgender respondents to capture the experiences of marginalized gender identities. We combined these categories because of small sample sizes and to allow comparison of associations with presumably cis-gendered men. However, this may not reflect the true identities of people within these categories. As such, we cannot disentangle the unique experiences of trans-men, trans-women, non-binary individuals, and cis-gendered women separately, for whom discrimination may manifest differently (51). For example, transgender people may experience rejection by their own family, or discrimination in healthcare settings (81), while women may experience disproportionately high expectations with respect to caregiving (82). This is also an issue for the grouping of racial/ethnic groups into marginalized vs. communities. Each marginalized group may have unique experiences, perceptions, or relationships with nature, reflecting distinct cultural, historical, and contextual backgrounds. Nevertheless, in the current analysis, because of low statistical power, we grouped a variety of racial/ethnic identities together. This grouping may have inadvertently obscured important relationships within each racial/ethnic group, which remains an important consideration for future research that explores questions such as the current one. Next, we did not have data on experiences of discrimination in parks or natural spaces, themselves. Instead, we used a measure of everyday discrimination that reflects experiences that may be recurrent but, in comparison to measures that explicitly assess other forms of interpersonal discrimination (e.g., traumatic experiences or major but acute events like wrongful termination or inhibition from accessing resources like housing), provides little insight into the context of severity or domain (83). Additionally, the GSS did not include the follow-up EDS question that asks respondents to report their perceptions for the reasons for inequitable treatment. Therefore, we cannot attribute experiences of discrimination to a specific identity. Also, the EDS does not capture all items that may be relevant to all forms of discrimination, nor do all respondents interpret the measure similarly. Previous work has shown that the interpretation of items on the EDS may vary according to sociodemographic or social-psychological factors and experiences (84–87). Additionally, some items within the EDS may not be as salient for all forms of discrimination. Other measures capturing manifestations of discrimination, such as gender or intersectional forms, for example, may also assess being treated with less courtesy or respect but may also ask about expectations of gender conformity, having your identity used as an insult, or experiencing sexual harassment (85, 88). This lack of specificity may mask important relationships that are salient to understand associations within specific identities. Further, the GSS defined nature as “parks, trails, and gardens”, only. This definition excludes many other types of green spaces, such as trees and forests, which may lead to considerable misreporting. We were also unable to determine whether reports of “nature experience” are related to experiences in public parks, or to wild forests or trails. The GSS also did not ascertain information on perceptions of green space quality or amenities, or safety features (e.g., lighting) in green spaces, which could mediate the association between discrimination and nature satisfaction. Finally, because we did not have access to small-area residential location data for the GSS respondents, we were unable to ascertain or adjust analyses for spatial measures of institutional or structural racism, such as present-day segregation or historical redlining, nor could we explore synergistic relationships between these measures with everyday discrimination.

Despite these limitations, we highlight several strengths of this work. To our knowledge, this is the first study to analyze the association of everyday discrimination with perceptions of nature experiences. Other strengths include the use of nationally representative survey data and our ability to adjust for several potential confounders, including employment status, age, and geographic region. In this study, we used a self-reported measure of everyday discrimination. Because this measure reflects respondents' perceptions of the world, it offers important insights into their personal interpretation of the lived experiences that may be intermediates on the pathway between structural determinants of oppression (e.g., historical redlining, medicalizing gender non-conformity) and experiences in nature (68, 69).

## Conclusion

There is increasing interest in creating opportunities for people to interact with nature; these efforts are often motivated by interest in promoting population health (89). In previous work, scholars have identified inequities in green or natural space availability across neighborhoods as a critical issue of distributive injustice and a consequence of historical, institutional, and structural systems of oppression (15, 16, 19, 20). Here, we moved beyond the question of nature availability to consider whether frequent discrimination is associated with perceptions of nature experiences. This work responds to recent calls for environmental researchers to make good on promises—made by non-profit, academic, professional societies, and governmental agencies in the wake of the racial reckoning that occurred in the U.S. in 2020—to interrogate racism and other systems and structures of social oppression that perpetuate disparities (40). Our results also highlight the importance of efforts that normalize the presence of historically marginalized groups in nature. For example, following widely publicized racist event in Central Park, New York, and in the wake of protests over police brutality against Black Americans, a group of scientists, nature lovers, and students established Black Birders Week. This is week-long series of events dedicated to promoting diversity in birding and nature experiences, and to dismantling perceptions that people of color do not belong in natural spaces (90). Results from our analysis suggest that people who experience more frequent everyday discrimination are less satisfied with their experiences in nature and with the amount of time spent there. Further research is needed to elucidate the attributions of experiences of discrimination, mechanisms underlying associations between everyday discrimination and nature experiences, and the intersecting and reinforcing impacts of different levels of societal marginalization on nature experiences. In addition, further research is needed to elucidate nature satisfaction as a causal intermediate on the pathway between discrimination and health outcomes. Results from this analysis point to the importance of considering the person-, cultural, and area-level contexts in which green spaces are situated and the need to dismantle systems of marginalization and oppression to build towards justice and equity when it comes to nature-based solutions.

## Data Availability

Publicly available datasets were analyzed in this study. This data can be found here: https://gss.norc.org/get-the-data.

## References

[1] Annerstedt van den Bosch M Depledge MH. Healthy people with nature in mind. BMC Public Health. (2015) 15:1–7. 10.1186/s12889-015-2574-8 26654879 PMC4676886

[2] Bennett G Cassin J Carroll N. Natural infrastructure investment and implications for the nexus: a global overview. Ecosyst Serv. (2016) 17:293–7. 10.1016/j.ecoser.2015.05.006

[3] Coventry PA Brown JE Pervin J Brabyn S Pateman R Breedvelt J Nature-based outdoor activities for mental and physical health: systematic review and meta-analysis. SSM Popul Health. (2021) 16:100934. 10.1016/j.ssmph.2021.100934 34646931 PMC8498096

[4] Twohig-Bennett C Jones A. The health benefits of the great outdoors: a systematic review and meta-analysis of greenspace exposure and health outcomes. Environ Res. (2018) 166:628–37. 10.1016/j.envres.2018.06.030 29982151 PMC6562165

[5] Richardson EA Pearce J Mitchell R Kingham S. Role of physical activity in the relationship between urban green space and health. Public Health. (2013) 127(4):318–24. 10.1016/j.puhe.2013.01.004 23587672

[6] Wan C Shen GQ Choi S. Underlying relationships between public urban green spaces and social cohesion: a systematic literature review. City Cult Soc. (2021) 24:100383. 10.1016/j.ccs.2021.100383

[7] Rook GA. Regulation of the immune system by biodiversity from the natural environment: an ecosystem service essential to health. Proc Natl Acad Sci U S A. (2013) 110(46):18360–7. 10.1073/pnas.1313731110 24154724 PMC3831972

[8] Suppakittpaisarn P Jiang X Sullivan WC. Green infrastructure, green stormwater infrastructure, and human health: a review. Curr Landsc Ecol Rep. (2017) 2:96–110. 10.1007/s40823-017-0028-y

[9] Schinasi LH Benmarhnia T De Roos AJ. Modification of the association between high ambient temperature and health by urban microclimate indicators: a systematic review and meta-analysis. Environ Res. (2018) 161:168–80. 10.1016/j.envres.2017.11.004 29149680

[10] Xu C Chen G Huang Q Su M Rong Q Yue W Can improving the spatial equity of urban green space mitigate the effect of urban heat islands? An empirical study. Sci Total Environ. (2022) 841:156687. 10.1016/j.scitotenv.2022.156687 35716736

[11] Dzhambov AM Markevych I Tilov B Arabadzhiev Z Stoyanov D Gatseva P Lower noise annoyance associated with GIS-derived greenspace: pathways through perceived greenspace and residential noise. Int J Environ Res Public Health. (2018) 15(7):1533. 10.3390/ijerph15071533 30029561 PMC6068578

[12] Markevych I Schoierer J Hartig T Chudnovsky A Hystad P Dzhambov AM Exploring pathways linking greenspace to health: theoretical and methodological guidance. Environ Res. (2017) 158:301–17. 10.1016/j.envres.2017.06.028 28672128

[13] Van den Berg AE Maas J Verheij RA Groenewegen PP. Green space as a buffer between stressful life events and health. Soc Sci Med. (2010) 70(8):1203–10. 10.1016/j.socscimed.2010.01.002 20163905

[14] Duncan DT Kawachi I Kum S Aldstadt J Piras G Matthews SA A spatially explicit approach to the study of socio-demographic inequality in the spatial distribution of trees across Boston neighborhoods. Spat Demogr. (2014) 2:1–29. 10.1007/BF03354902 29354668 PMC5771436

[15] Casey JA James P Cushing L Jesdale BM Morello-Frosch R. Race, ethnicity, income concentration and 10-year change in urban greenness in the United States. Int J Environ Res Public Health. (2017) 14(12):1546. 10.3390/ijerph14121546 PMC5750964 29232867

[16] Jesdale BM Morello-Frosch R Cushing L. The racial/ethnic distribution of heat risk-related land cover in relation to residential segregation. Environ Health Perspect. (2013) 121(7):811–7. 10.1289/ehp.1205919 23694846 PMC3701995

[17] Jennings V Johnson Gaither C. Approaching environmental health disparities and green spaces: an ecosystem services perspective. Int J Environ Res Public Health. (2015) 12(2):1952–68. 10.3390/ijerph120201952 25674782 PMC4344703

[18] Low S. Public space and diversity: distributive, procedural and interactional justice for parks. In: Stevenson D, Young G, editor. The Routledge Research Companion to Planning and Culture. London: Routledge (2013). p. 295–310.

[19] Nardone A Rudolph KE Morello-Frosch R Casey JA. Redlines and greenspace: the relationship between historical redlining and 2010 greenspace across the United States. Environ Health Perspect. (2021) 129(1):17006. 10.1289/EHP7495 33502254 PMC7839347

[20] Schinasi LH Kanungo C Christman Z Barber S Tabb L Headen I. Associations between historical redlining and present-day heat vulnerability housing and land cover characteristics in Philadelphia, PA. J Urban Health. (2022) 99(1):134–45. 10.1007/s11524-021-00602-6 35076872 PMC8866576

[21] Edwards FL Thompson GB. The legal creation of raced space: the subtle and ongoing discrimination created through Jim Crow laws. Berkeley J Afr Am L Pol'y. (2010) 12:145–68. https://heinonline.org/HOL/P?h=hein.journals/afamlpol12&i=149

[22] Lee KJ Fernandez M Scott D Floyd M. Slow violence in public parks in the US: can we escape our troubling past? Soc Cult Geogr. 24(7):1185–202. 10.1080/14649365.2022.2028182

[23] Daniel R. Understanding our environment requires an indigenous worldview. Eos. (2019) 100. 10.1029/2019EO137482

[24] Hoover F-A Lim TC. Examining privilege and power in US urban parks and open space during the double crises of antiblack racism and COVID-19. Socioecol Pract Res. (2021) 3(1):55–70. 10.1007/s42532-020-00070-3 34778707 PMC7684571

[25] Gripper AB. Practices of care and relationship-building: a qualitative analysis of urban agriculture’s impacts on black people’s agency and wellbeing in Philadelphia. Int J Environ Res Public Health. (2023) 20(6):4831. 10.3390/ijerph20064831 36981740 PMC10049229

[26] Reese AM. “We will not perish; we’re going to keep flourishing”: race, food access, and geographies of self-reliance. Antipode. (2018) 50(2):407–24. 10.1111/anti.12359

[27] Sue DW. Microaggressions in Everyday Life: Race, Gender, and Sexual Orientation. Hoboken: John Wiley & Sons (2010).

[28] Perry SP Hardeman R Burke SE Cunningham B Burgess DJ van Ryn M. The impact of everyday discrimination and racial identity centrality on African American medical student well-being: a report from the medical student CHANGE study. J Racial Ethn Health Disparities. (2016) 3(3):519–26. 10.1007/s40615-015-0170-3 27294743 PMC4911311

[29] Chen D Yang T-C. The pathways from perceived discrimination to self-rated health: an investigation of the roles of distrust, social capital, and health behaviors. Soc Sci Med. (2014) 104:64–73. 10.1016/j.socscimed.2013.12.021 24581063 PMC4048349

[30] Davis J. Black faces, black spaces: rethinking African American underrepresentation in wildland spaces and outdoor recreation. Environ Plan E Nat Space. (2019) 2(1):89–109. 10.1177/2514848618817480

[31] Evenson KR Jones SA Holliday KM Cohen DA McKenzie TL. Park characteristics, use, and physical activity: a review of studies using SOPARC (system for observing play and recreation in communities). Prev Med. (2016) 86:153–66. 10.1016/j.ypmed.2016.02.029 26946365 PMC4837088

[32] Derose KP Han B Williamson S Cohen DA. Gender disparities in park use and physical activity among residents of high-poverty neighborhoods in Los Angeles. Womens Health Issues. (2018) 28(1):6–13. 10.1016/j.whi.2017.11.003 29241943 PMC5753770

[33] Sreetheran M Van Den Bosch CCK. A socio-ecological exploration of fear of crime in urban green spaces–a systematic review. Urban For Urban Green. (2014) 13(1):1–18. 10.1016/j.ufug.2013.11.006

[34] Basu S Nagendra H. Perceptions of park visitors on access to urban parks and benefits of green spaces. Urban For Urban Green. (2021) 57:126959. 10.1016/j.ufug.2020.126959

[35] Lampe TM Reisner SL Schrimshaw EW Radix A Mallick R Harry-Hernandez S Navigating stigma in neighborhoods and public spaces among transgender and nonbinary adults in New York City. Stigma Health. (2020) 5(4):477. 10.1037/sah0000219

[36] Rood BA Reisner SL Surace FI Puckett JA Maroney MR Pantalone DW. Expecting rejection: understanding the minority stress experiences of transgender and gender-nonconforming individuals. Transgender Health. (2016) 1(1):151–64. 10.1089/trgh.2016.0012 29159306 PMC5685272

[37] Ransom J. Amy cooper faces charges after calling police on black bird-watcher. The New York Times. (2020). https://www.nytimes.com/2020/07/06/nyregion/amy-cooper-false-report-charge.html (Accessed May 17, 2024).

[38] Harnois CE Bastos JL Shariff-Marco S. Intersectionality, contextual specificity, and everyday discrimination: assessing the difficulty associated with identifying a main reason for discrimination among racial/ethnic minority respondents. Sociol Methods Res. (2020) 51(3):983–1013. 10.1177/0049124120914929

[39] Moody MD. Vicarious experiences of major discrimination and the life satisfaction of black and white adults from a community sample. J Happiness Stud. (2022) 23(6):2725–43. 10.1007/s10902-022-00532-3

[40] Payne-Sturges DC Gee GC Cory-Slechta DA. Confronting racism in environmental health sciences: moving the science forward for eliminating racial inequities. Environ Health Perspect. (2021) 129(5):55002. 10.1289/EHP8186 33945300 PMC8096378

[41] Williams DR Lawrence JA Davis BA. Racism and health: evidence and needed research. Annu Rev Public Health. (2019) 40:105–25. 10.1146/annurev-publhealth-040218-043750 30601726 PMC6532402

[42] Anderson KF. Diagnosing discrimination: stress from perceived racism and the mental and physical health effects. Sociol Inq. (2013) 83(1):55–81. 10.1111/j.1475-682X.2012.00433.x

[43] Hosler AS Kammer JR Cong X. Everyday discrimination experience and depressive symptoms in urban black, Guyanese, Hispanic, and white adults. J Am Psychiatr Nurses Assoc. (2019) 25(6):445–52. 10.1177/1078390318814620 30569835

[44] Smith TW. General Social Surveys, 1972–2018 [Machine-Readable Data File]/Principal Investigator, Smith, Tom W.; Co-Principal Investigators, Michael Davern, Jeremy Freese, and Stephen Morgan; Sponsored by National Science Foundation. Chicago: NORC (2018). p. 7990–5.

[45] Smith TW Son J. Transgender and Alternative Gender Measurement on the 2018 General Social Survey. Chicago, IL: NORC at the University of Chicago (2019).

[46] Davern M, Bautista R, Freese J, Herd P, Morgan SL. General Social Survey 2018 Ballot 3. NORC, editor. Chicago (2018).

[47] Krieger N. Racial and gender discrimination: risk factors for high blood pressure? Soc Sci Med. (1990) 30(12):1273–81. 10.1016/0277-9536(90)90307-E 2367873

[48] Krieger N Sidney S. Racial discrimination and blood pressure: the CARDIA study of young black and white adults. Am J Public Health. (1996) 86(10):1370–8. 10.2105/AJPH.86.10.1370 8876504 PMC1380646

[49] Brondolo E Brady ver Halen N Pencille M Beatty D Contrada RJ. Coping with racism: a selective review of the literature and a theoretical and methodological critique. J Behav Med. (2009) 32:64–88. 10.1007/s10865-008-9193-0 19127420 PMC3258496

[50] Pyke KD. What is internalized racial oppression and why don't we study it? Acknowledging racism’s hidden injuries. Sociol Perspect. (2010) 53(4):551–72. 10.1525/sop.2010.53.4.551

[51] Crosby F. The denial of personal discrimination. Ame Behav Sci. (1984) 27(3):371–86. 10.1177/000276484027003008

[52] McNutt L-A Wu C Xue X Hafner JP. Estimating the relative risk in cohort studies and clinical trials of common outcomes. Am J Epidemiol. (2003) 157(10):940–3. 10.1093/aje/kwg074 12746247

[53] Zou G. A modified poisson regression approach to prospective studies with binary data. Am J Epidemiol. (2004) 159(7):702–6. 10.1093/aje/kwh090 15033648

[54] Harrison S Quigley MA Fellmeth G Stein A Alderdice F. The impact of the COVID-19 pandemic on postnatal depression: analysis of three population-based national maternity surveys in England (2014–2020). Lancet Reg Health Eur. (2023) 30:100654. 10.1016/j.lanepe.2023.100654. [Epub ahead of print]. 37363795 PMC10183799

[55] Scheim AI Perez-Brumer AG Bauer GR. Gender-concordant identity documents and mental health among transgender adults in the USA: a cross-sectional study. Lancet Public Health. (2020) 5(4):e196–203. 10.1016/S2468-2667(20)30032-3 32192577 PMC9912749

[56] Howe CJ Bailey ZD Raifman JR Jackson JW. Recommendations for using causal diagrams to study racial health disparities. Am J Epidemiol. (2022) 191(12):1981–9. 10.1093/aje/kwac140 35916384 PMC10144617

[57] Bailey ZD Feldman JM Bassett MT. How structural racism works—racist policies as a root cause of US racial health inequities. N Engl J Med. (2021) 384(8):768–73. 10.1056/NEJMms2025396 33326717 PMC11393777

[58] Krieger N. Genders, sexes, and health: what are the connections—and why does it matter? Int J Epidemiol. (2003) 32(4):652–7. 10.1093/ije/dyg156 12913047

[59] Kaufman JS MacLehose RF. Which of these things is not like the others? Cancer. (2013) 119(24):4216–22. 10.1002/cncr.28359 24022386 PMC4026206

[60] R Core Team. R: A Language and Environment for Statistical Computing. Vienna, Austria: R Foundation for Statistical Computing (2021). Available online at: https://www.R-project.org/

[61] Lumley T. Package “Survey”. (2020). Available online at: https://cran.r-project.org (Access February 14, 2024).

[62] Kaplan S. The restorative environment: nature and human experience. In: Relf D, editor. The Role of Horticulture in Human Well Being and Social Development. Portland, OR: Timber Press (1992). p. 134–42.

[63] Zhang N Gereke J Baldassarri D. Everyday discrimination in public spaces: a field experiment in the Milan Metro. Eur Sociol Rev. (2022) 38(5):679–93. 10.1093/esr/jcac008

[64] Jones CP. Levels of racism: a theoretic framework and a gardener's tale. Am J Public Health. (2000) 90(8):1212–5. 10.2105/AJPH.90.8.1212 10936998 PMC1446334

[65] Reskin B. The race discrimination system. Annu Rev Sociol. (2012) 38:17–35. 10.1146/annurev-soc-071811-145508

[66] Williams DR Lawrence JA Davis BA Vu C. Understanding how discrimination can affect health. Health Serv Res. (2019) 54:1374–88. 10.1111/1475-6773.13222 31663121 PMC6864381

[67] Lee KJ Scott D. Bourdieu and African Americans’ park visitation: the case of Cedar Hill state park in Texas. Leis Sci. (2016) 38(5):424–40. 10.1080/01490400.2015.1127188

[68] Crawford D Timperio A Giles-Corti B Ball K Hume C Roberts R Do features of public open spaces vary according to neighbourhood socio-economic status? Health Place. (2008) 14(4):889–93. 10.1016/j.healthplace.2007.11.002 18086547

[69] Weiss CC Purciel M Bader M Quinn JW Lovasi G Neckerman KM Reconsidering access: park facilities and neighborhood disamenities in New York City. J Urban Health. (2011) 88:297–310. 10.1007/s11524-011-9551-z 21360245 PMC3079030

[70] Kamel AA Ford PB Kaczynski AT. Disparities in park availability, features, and characteristics by social determinants of health within a US–Mexico border urban area. Prev Med. (2014) 69:S111–3. 10.1016/j.ypmed.2014.10.001 25451326

[71] Engelberg JK Conway TL Geremia C Cain KL Saelens BE Glanz K Socioeconomic and race/ethnic disparities in observed park quality. BMC Public Health. (2016) 16:1–11. 10.1186/s12889-016-3055-4 27176854 PMC4866396

[72] McKenzie TL Moody JS Carlson JA Lopez NV Elder JP. Neighborhood income matters: disparities in community recreation facilities, amenities, and programs. J Park Recreat Admi. (2013) 31(4):12–22. ; . 25006598 PMC4082954

[73] Suminski RR Connolly EK May LE Wasserman J Olvera N Lee RE. Park quality in racial/ethnic minority neighborhoods. Environ Justice. (2012) 5(6):271–8. 10.1089/env.2012.0013

[74] Zhang Y Van den Berg AE Van Dijk T Weitkamp G. Quality over quantity: contribution of urban green space to neighborhood satisfaction. Int J Environ Res Public Health. (2017) 14(5):535. 10.3390/ijerph14050535 28509879 PMC5451986

[75] Gordon MT Riger S LeBailly RK Heath L. Crime, women, and the quality of urban life. Signs J Women Cult Soc. (1980) 5(S3):S144–60. 10.1086/495716

[76] Tandogan O Ilhan BS. Fear of crime in public spaces: from the view of women living in cities. Procedia Eng. (2016) 161:2011–8. 10.1016/j.proeng.2016.08.795

[77] Madge C. Public parks and the geography of fear. Tijdschr Econ Soc Ge. (1997) 88(3):237–50. 10.1111/j.1467-9663.1997.tb01601.x

[78] Kondo MC Clougherty JE Hohl BC Branas CC. Gender differences in impacts of place-based neighborhood greening interventions on fear of violence based on a cluster-randomized controlled trial. J Urban Health. (2021) 98:812–21. 10.1007/s11524-021-00580-9 34750735 PMC8688630

[79] Richardson M McEwan K Maratos F Sheffield D. Joy and calm: how an evolutionary functional model of affect regulation informs positive emotions in nature. Evol Psychol Sci. (2016) 2:308–20. 10.1007/s40806-016-0065-5

[80] McEachan RR Yang TC Roberts H Pickett KE Arseneau-Powell D Gidlow CJ Availability, use of, and satisfaction with green space, and children’s mental wellbeing at age 4 years in a multicultural, deprived, urban area: results from the born in Bradford cohort study. Lancet Planet Health. (2018) 2(6):e244–54. 10.1016/S2542-5196(18)30119-0 29880156

[81] Hughto JMW Reisner SL Pachankis JE. Transgender stigma and health: a critical review of stigma determinants, mechanisms, and interventions. Soc Sci Med. (2015) 147:222–31. 10.1016/j.socscimed.2015.11.010 26599625 PMC4689648

[82] Krieger N. Measures of racism, sexism, heterosexism, and gender binarism for health equity research: from structural injustice to embodied harm-an ecosocial analysis. Annu Rev Public Health. (2019) 41:37–62. 10.1146/annurev-publhealth-040119-094017 31765272

[83] Williams DR. Improving the measurement of self-reported racial discrimination: challenges and opportunities. In: Alvarez AN, Liang CTH, Neville HA, editors. The Cost of Racism for People of Color: Contextualizing Experiences of Discrimination. American Psychological Association (2016). p. 55–83. 10.1037/14852-004

[84] Harnois CE. What do we measure when we measure perceptions of everyday discrimination? Soc Sci Med. (2022) 292:114609. 10.1016/j.socscimed.2021.114609 34894458

[85] Harnois CE Ifatunji M. Gendered measures, gendered models: toward an intersectional analysis of interpersonal racial discrimination. Ethn Racial Stud. (2011) 34(6):1006–28. 10.1080/01419870.2010.516836

[86] Lewis TT Yang FM Jacobs EA Fitchett G. Racial/ethnic differences in responses to the everyday discrimination scale: a differential item functioning analysis. Am J Epidemiol. (2012) 175(5):391–401. 10.1093/aje/kwr287 22306556 PMC3282874

[87] Harnois CE Bastos JL Campbell ME Keith VM. Measuring perceived mistreatment across diverse social groups: an evaluation of the everyday discrimination scale. Soc Sci Med. (2019) 232:298–306. 10.1016/j.socscimed.2019.05.011 31121440

[88] Scheim AI Bauer GR. The intersectional discrimination Index: development and validation of measures of self-reported enacted and anticipated discrimination for intercategorical analysis. Soc Sci Med. (2019) 226:225–35. 10.1016/j.socscimed.2018.12.016 30674436

[89] Ulmer JM Wolf KL Backman DR Tretheway RL Blain CJ O’Neil-Dunne JP Multiple health benefits of urban tree canopy: the mounting evidence for a green prescription. Health Place. (2016) 42:54–62. 10.1016/j.healthplace.2016.08.011 27639106

[90] National Audubon Society. Black Birders Week 2024. (2024). Available online at: https://www.audubon.org/black-birders-week (Accessed April 19, 2024).

