# Supplementary material for: Everyday discrimination and satisfaction with nature experiences
Source: Front Epidemiol. 2024 May 30;4:1212114. doi: 10.3389/fepid.2024.1212114 (PMC11169619; doi:10.3389/fepid.2024.1212114)
Supplement: Supplementary file 1 [file Table1.docx]

Supplementary Material

Everyday discrimination and satisfaction with nature experiences

Leah H Schinasi*, Jourdyn Lawrence

*** Correspondence:** Corresponding Author: lhs36@drexel.edu

Supplemental Table. Comparison of AIC statistics, under different scenarios where the models were run with the reverse coded, median centered everyday discrimination score parameterized as a natural cubic spline term with 3 degrees of freedom (DF) or 2 DF, or as a linear term. These models were run and compared to identify optimal coding for the everyday discrimination score variable.

|  | AIC statistic | | |
| --- | --- | --- | --- |
|  | 3 DF | 2 DF | LINEAR |
| **Do not spend as much time as you want in nature** | 4538.8 | 4535.2 | 4531.1 |
| **Not satisfied with experience in nature** | 4488.8 | 4488.7 | 4489.6 |
| **Do not spend at least one day per week in nature** | 4593.5 | 4590.8 | 4586.7 |
