# Supplementary material for: Everyday discrimination and satisfaction with nature experiences
Source: Front Epidemiol. 2024 May 30;4:1212114. doi: 10.3389/fepid.2024.1212114 (PMC11169619; doi:10.3389/fepid.2024.1212114)

Supplementary Material

Everyday discrimination and satisfaction with nature experiences

Leah H Schinasi*, Jourdyn Lawrence

*** Correspondence:** Corresponding Author: [lhs36@drexel.edu](mailto:lhs36@drexel.edu)

Supplemental Figure. Directed Acyclic Graph depicting hypothesized relationships between everyday discrimination, nature experiences, and covariates. This figure was used to identify a minimally sufficient adjustment set for the models.


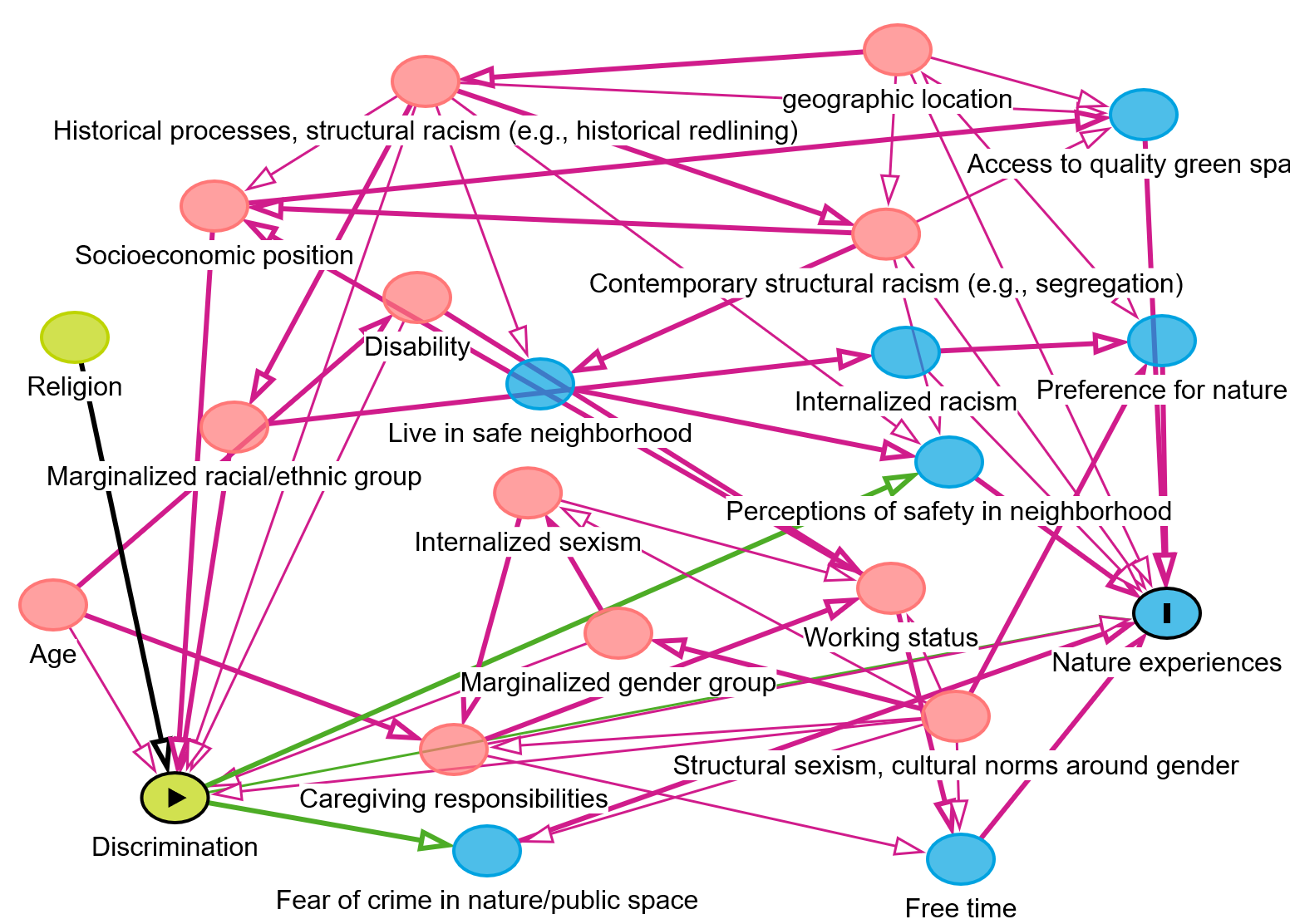

Supplement: Supplementary file 2 [file Datasheet1.docx]
